# Editorial: Novel technologies applied to flavoromics and sensory evaluation of foods

**DOI:** 10.3389/fnut.2024.1544709

**Published:** 2025-01-14

**Authors:** Geraldine M. Dowling, Michel Aliani

**Affiliations:** ^1^Department of Life Sciences, School of Science, Atlantic Technological University, Sligo, Ireland; ^2^Cameron Forensic Medical Sciences, William Harvey Research Institutes, Barts and The London School of Medicine and Dentistry, Queen Mary University of London, London, United Kingdom; ^3^Department of Analytical, Environmental and Forensic Sciences, School of Cancer and Pharmaceutical Sciences, Faculty of Life Sciences and Medicine, King's College London, London, United Kingdom; ^4^Department of Food and Human Nutritional Sciences, Faculty of Agriculture and Food Sciences, University of Manitoba, Winnipeg, MB, Canada; ^5^The Division of Neurodegenerative Disorders, St. Boniface Hospital Research Centre, Winnipeg, MB, Canada

**Keywords:** flavoromics, sensory analysis, nuclear magnetic resonance (NMR) spectroscopy, chromatography, mass spectrometry (MS), food, analytical techniques, chemometrics

Flavoromics, an emerging field that combines chemometrics and progressive analytical techniques, aims to understand the complex processes behind flavor formation in foods. By using data from diverse samples, it helps address challenges such as distinguishing similar food products and ensuring authenticity.

## Flavoromics

Flavoromics has the potential to transform various disciplines by providing a deeper understanding of the chemical compounds responsible for flavor. It can enhance food quality, support the development of functional foods and beverages and expand product formulations across industries. In the herbal and medicinal fields, flavoromics helps isolate bioactive compounds, supporting the discovery of new therapeutic properties. Flavoromics has promising applications in medicine, particularly by enhancing the sensory experience of food, supporting personalized nutrition, and hypothetically aiding in therapeutic and pharmaceutical applications. Its interdisciplinary applications extend to agriculture, biotechnology, and even environmental sciences, offering valuable insights for improving flavors, health benefits, and sustainability in diverse products and processes into the future.

Sensory evaluation, a key component of flavoromic studies, is defined as the scientific discipline dedicated to assessing the eating quality of food by systematically measuring human responses to its sensory attributes, including aroma, appearance, texture and flavor. This field relies on both qualitative and quantitative methods to capture how consumers perceive and interact with food products.

While sensory evaluation provides direct insights into human perception, it is often complemented by analytical and instrumental techniques. These tools are essential for identifying and quantifying the formation of specific chemical compounds responsible for the sensory characteristics detected by the olfactory (smell) and gustatory (taste) systems.

In complex food systems, the generation of flavor compounds is influenced by a wide range of chemical and biochemical reactions. These reactions can involve the breakdown of proteins, carbohydrates, and fats, as well as the interaction of enzymes, heat and pH conditions during food processing. Understanding these intricate processes is crucial for optimizing product quality, enhancing sensory appeal and ensuring consistency in food production. Ultimately, sensory evaluation, combined with analytical techniques, provides a holistic approach to understanding and improving the overall sensory experience of food.

Advanced analytical techniques, including chromatography, mass spectrometry and spectroscopy, play a crucial role in dissecting the complex chemical profiles that define the flavor, aroma, and texture of food products. These methods enable precise identification and quantification of volatile and non-volatile compounds, providing a detailed understanding of the molecular components that contribute to sensory perception.

Several original research manuscripts presented here offer some key sensory methods and/or analytical tools used in flavoromics studies of orange juice, raw and pasteurized honey, tilapia by products, Fritillaria, rhubarb, steamed beef with rice flour, herbs, Zheng'an Bai tea and Saskatoon berry fortified Greek style frozen yogurt. Five of these studies included sensory evaluation methods and a range of instrumental measurements such as gas chromatography, mass spectrometry, nuclear magnetic resonance (NMR) spectroscopy, and electronic nose and electronic tongue.

Kardas, Kiciak et al., assessed the color of orange juice in the context of dietitian's food preferences. The research demonstrated that dietitian's preferred bright juices with a vibrant orange hue while product packaging influences the dietitian choice regardless of the content.

Ren et al., identified 16 novel salty peptides from hydrolysates of tilapia by products by batch molecular docking. They were predominately salty with a threshold of 0.256–0.379 mmol/L with some sourness and astringency where HLDDALR had the highest salty intensity.

Kardas, Staśkiewicz-Bartecka et al., studied the quality of selected raw and pasteurized honeys based on their sensory profiles and consumer preferences demonstrating consumer preference for the taste of pasteurized honeys.

Dai et al., explored the rapid detection of Fritillaria using gas chromatography ion mobility spectrometry to identify 67 volatile organic compounds which may be used for identification and authenticity determination of varieties of Fritillaria.

Liu T. et al., optimized a traditional method of rhubarb processing by combining flavor analysis with anthraquinone content. The results showed SDR-6 and SDR-9 in terms of smell, taste and composition indicating that the steaming and sun-drying cycles can be reduced from 9 to 6.

Wang et al., characterized the flavor profile of steamed beef with rice flour (SBD) using gas chromatography ion mobility spectrometry combined with electronic nose and tongue offering valuable insights into the industrial scale production and flavor regulation of SBD products.

Wu et al., developed an ic-CLEIA for precise detection of 3-CQA in herbs and patent medicines ensuring quality control and therapeutic efficiency with significant potential for diverse therapeutic milieus and applications.

Liu L. et al., used targeted metabolomics and SPME-GC-MS analysis to reveal the quality characteristics of non-volatile/volatile compounds in Zheng'an Bai tea which provided the foundation for further processing improvement.

Ryland et al., employed a flavoromics approach to investigate the effect of saskatoon berry powder on sensory attributes, acceptability, volatile components and electronic nose responses of a low-fat frozen yogurt with potential to fortified dairy products.

Together, these advanced technologies and studies facilitate a deeper understanding of the molecular basis of flavor and enable the development of innovative, high-quality food products tailored to consumer preferences. By integrating traditional analytical techniques with AI-driven data analysis, flavoromics provides a powerful framework for advancing sensory science and food innovation.

We hope this Research Topic will serve as a potential template for food scientists, researchers, food, and nutraceutical companies. Flavoromics has diverse applications. It could be used in medicine to improve the taste of medicines, particularly liquid medications or those intended for children or people with swallowing difficulties. By enhancing the flavor profile of medications, patient compliance and overall medication adherence can be improved. In addition, understanding the chemical components that contribute to flavor, food scientists can develop appealing, nutritious foods to inspire better dietary habits. The use of flavoromics in conjunction with genomics can help tailor dietary recommendations based on an individual's genetic makeup and flavor preferences. This can potentially lead to personalized nutrition plans that enhance health outcomes and prevent disease. More research in flavoromics can also contribute to the development of functional foods developed to prevent or manage conditions such as cardiovascular disease, diabetes, and obesity by understanding how specific flavor compounds influence biological systems, like metabolism and blood sugar regulation.

